# Social cohesion and attitudinal changes toward migration: A longitudinal perspective amid the COVID-19 pandemic

**DOI:** 10.3389/fsoc.2022.1009567

**Published:** 2023-01-23

**Authors:** Juan Carlos Castillo, Macarena Bonhomme, Daniel Miranda, Julio Iturra

**Affiliations:** ^1^Department of Sociology, University of Chile, Santiago, Chile; ^2^Faculty of Social Sciences and Humanities, Universidad Autónoma de Chile, Santiago, Chile; ^3^MIDE UC, Pontificia Universidad Católica de Chile, Santiago, Chile; ^4^Bremen International Graduate School of Social Sciences, Bremen, Germany

**Keywords:** migration, social cohesion, conviviality, threat, identity, Chile, COVID-19, South-South migration

## Abstract

The COVID-19 pandemic has impacted social interactions and coexistence around the globe in dimensions that go far beyond health issues. In the case of the Global South, the pandemic has developed along with growing South-South migratory movements, becoming another key factor that might reinforce social conflict in increasingly multicultural areas as migrants have historically served as “scapegoats” for unexpected crises as a way to control and manage diversity. Chile is one of the main destination countries for migrants from the Latin American and Caribbean region, and COVID-19 outbreaks in migrant housing have intensified discrimination. In such a context, there is a need for understanding how the pandemic has potentially changed the way non-migrants perceive and interact with migrant neighbors. Drawing on the national social cohesion panel survey study ELSOC (2016–2021, *N* = 2,927) the aim is to analyze the changes in non-migrants' attitudes toward migrants—related to dimensions of social cohesion—over the last years and their relation with individual status and territorial factors. We argue that social cohesion in increasingly multicultural societies is partially threatened in times of crisis. The results indicate that after the pandemic, convivial attitudes toward Latin American migrants decreased. Chileans started perceiving them more negatively, particularly those respondents with lower educational levels and who live in increasingly multicultural neighborhoods with higher rates of migrant residents.

## Introduction

The sanitary and economic crises produced by the COVID-19 pandemic have generated radical changes in different dimensions of society. Given that the pandemic has occurred along with several migratory movements around the world, one question that emerges is to what extent these mobilities have impacted social cohesion since the outbreak of COVID-19. One aspect to consider is that there is a complex historical relationship between migration, ethnicity/“race,” and contagious diseases (Briggs, 2005; Ahmad and Bradby, 2007; Kraut, 2010; Cecchi, 2019; von Unger et al., 2019), as there has been an association between vulnerable social groups and the way they inhabit urban spaces in times of epidemics (see Craddock, 1995; Sawchuk and Burke, 1998). Several pandemics have been blamed on underprivileged groups (Sennett, 1997; Meza, 1999), such as migratory, ethnic minorities and even low-income national groups (the urban “poor”) in the context of growing urbanization (Connolly et al., 2020), who are either perceived or fabricated as the “other” and potentially associated with contagious diseases. In the context of growing migratory movements, countries historically have used migrant communities as tools to enable their own political agenda in the face of health or economic crises (Cecchi, 2019). Constructing scapegoats in an “other” becomes a way societies control and manage what they consider “diverse” (Ahmad and Bradby, 2007; Cecchi, 2019). For instance, epidemiologists associated the spread of the SARS virus with the cultural practices of southern Chinese people (Mason, 2015, p. 507), which were deeply racialized.

Amid the ongoing COVID-19 sanitary crises, Chile^1^, as one of the countries with major migratory flows from the Latin American and the Caribbean (LAC) region (OIM, 2018), becomes a relevant case study to understand the impact of this pandemic in the Global South concerning the coexistence between Chileans and migrants in increasingly multicultural neighborhoods. Over the past decades, the number of migrants in Chile has risen significantly. While in 2002, migrants residing in Chile comprised only 1.3% of the total population (INE, 2018), by the end of 2020 they accounted for more than 8%, according to the latest estimates (INE and DEM, 2021). These migrations fluxes have been predominately South-South: mainly from Venezuela (30.7%), followed by Peru (16.3%), Haiti (12.5%), Colombia (11.4%), and Bolivia (8.5%), among other countries (INE and DEM, 2021). The vast majority of migrants arrived between 2010 and 2017 (66.7%), which constitutes an unprecedented migration compared to previous years (INE, 2017) and with growing irregular mobilities (SJM, 2022). In Chile's capital, most migrants live in low-income and segregated areas, inhabiting collective housing or *campamentos* (squatter settlements) (Pérez and Palma, 2021) that are characterized by the precarious and overcrowded living conditions due to the excessive profiteering from Chileans and long-time migrants (Bonhomme, 2021). These issues that stem from major political, economic, and social processes have led to social conflict, reinforcing racism, especially in low-income neighborhoods (Bonhomme, 2021).

While some studies have analyzed Chileans' perceptions toward migration and intercultural relations (see González et al., 2010; Thayer et al., 2013; Bonhomme, 2021, 2022), little research has focused on the ways in which these perceptions and interactions might have changed in times of crises. Nor has it looked at the entangled relationship between the COVID-19 pandemic, migration, and social cohesion. The aim of this paper is to assess Chileans' attitudes toward South-South migration in order to understand how this aspect of social cohesion has been impacted due to the pandemic COVID-19 and its aftermath. Social cohesion has been defined as a multidimensional concept that usually includes aspects such as common goals and values, a sense of belonging and identity, tolerance and respect for diversity, interpersonal and institutional trust, civic cooperation, active participation, and law-abiding behavior (Green and Janmaat, 2011). In the present study, we focus on particular aspects of social cohesion that is more closely related to our research problem which deals with migration in the pandemic context: conviviality, identity and perceived threat. Conviviality refers to the process of multi-ethnic cohabitation and interaction in a territory (Gilroy, 2004), and it is understood here as a friendly coexistence with neighbors. Identity deals with the perception of moral differences (or similarities), values, customs, beliefs, or cultural practices (Stephan et al., 2000), whereas the perceived threat is understood as non-migrants' worries about the potential impact on unemployment due to migration. Within this framework, this article aims to contribute to the understanding of the attitudinal changes in non-migrants toward the most prominent Latin American and Caribbean migratory groups living in Chile in the context of major economic, social, health, and political crises between 2016 and 2021.

## Attitudes toward migration in pandemic contexts

Migrants have been historically seen as a potential threat and stigmatized as “disease carriers” despite evidence to the contrary (Kraut, 2010). One example is the influenza pandemic of 1918–1919 in the US, which coincided with the increased mobility of migrants (Southern Italians and Eastern European Jews), who were seen as a threat to society. Kraut (2010) unveils that though there was no general association of migrants as the cause of the pandemic, they did face prejudice regarding health. Because of cultural differences and the rural origins of most, as well as their overcrowded dwelling places, they were identified as facilitators of contagion. However, poverty was a key factor, since the congested living conditions, long working hours, and malnourishment of newcomer migrants made them more vulnerable (2010, p. 127). In addition, the linguistic barrier (for some migrants) encumbered their compliance with state-mandated measures regarding the pandemic (2010). In the collective imaginaries, however, people's values, behaviors, and customs that differ from mainstream society's morals have been associated with susceptibility to infectious diseases. In that sense, since infectious disease outbreaks constitute threatening events, people usually require “collective symbolic coping” (Eicher and Bangerter, 2015), which means representing the outgroup's practices as immoral.

Analyses suggest that contemporary processes of urbanization may increase vulnerability to the spread of infectious diseases (Ali and Keil, 2006; Roberts, 2009; Connolly et al., 2020). Ali and Keil (2006), regarding the SARS outbreak in Toronto, reveal how spatial factors have historically impacted negatively racialized communities. Deprived neighborhoods have a direct effect on people's opportunities and can reinforce social exclusion (Atkinson and Kintrea, 2001; Harvey, 2008), not only in terms of access to resources but also in terms of the stigmatized perceptions regarding residents, that affect the quality of life and especially employment and health (Atkinson and Kintrea, 2001; Buck, 2001). Other studies show that in the context of pandemics, non-migrants discriminate against (perceived) non-white communities, perpetuating a discourse of inferiority that translated into a perception of weaker health (Roberts, 2009). In this line, recent evidence confirms that the COVID-19 outbreak, once again, boosted anti-immigrant sentiment against Chinese residents around the globe (Chan and Montt, 2020; Tessler et al., 2020). In the case of Chile, the pandemic has reinforced stereotypes of migrant communities, especially Afro-descendant migrants who were targeted as threats. For instance, in digital spaces Chileans portrayed Haitian migrants as “filthy” and disease carriers, reproducing anti-black racism and reinforcing an anti-immigrant sentiment that aimed to control migratory mobilities into Chile (Bonhomme and Alfaro, 2022).

### Conviviality, identity and threat in the context of growing migration

In order to grasp social cohesion and the way it might have changed over the years, we focused on three dimensions: conviviality, identity, and threat. Following Gilroy (2004, p. 11), we use the term conviviality to refer to the process of multi-ethnic cohabitation and interaction in a territory. Gilroy's theorization of conviviality, from a postcolonial perspective, allows challenging the notion of integration and its normative canons of nationally-based identities and culturalism, to embrace contemporary forms of multiculturalism (Valluvan, 2016). Gilroy (2004, p. 105) calls for an interaction whereby the difference among identities becomes “politically unremarkable” and where perceived “racial” differences are not feared. In that sense, it implies that people need to have the capacity to be at ease with the presence of diversity (Valluvan, 2016). However, Redclift et al. (2022, p. 14) argue that the people who actually do convivial work on a daily basis are those considered to be inferior within a white normativity. This is what the authors (2022, p. 2) call the “burden of conviviality”. This study in the UK shows that negatively racialized migrants navigate the fact of being “Othered” through different ways of putting at ease those who are not racialized as “different” so that surviving this unevenly distributed burden of conviviality meant “disappearing into normative whiteness” (Redclift et al., 2022, p. 14). In that sense, a convivial culture does not mean tolerance or the end of racism in multicultural neighborhoods. Conviviality is in effect contiguous to processes of ethnic conflict (Valluvan, 2016). In the case of Chile, similar to other Latin American countries (Loveman, 2009), measuring this concept is particularly interesting as it has historically taken whiteness for granted and Chileans tend to negatively racialize LAC migrants and perceive them as “inferior” based on racist logics (Bonhomme, 2022). Even though we acknowledge the complexities behind the term conviviality, considering that this study's survey data only focuses on Chilean citizens, we will measure it as an attitude toward a constructed “other”. In this case, toward LAC migrants. This will allow us to measure at least one side of this process of multi-ethnic cohabitation, that is, from the non-migrants' perspective. Therefore, a convivial attitude will be understood here as the individuals' ability to interact and have a friendly coexistence with those they consider ethnically different from themselves.

Besides conviviality, the literature on the development of social cohesion attitudes in migratory contexts has focused on other essential aspects to understand the phenomenon. Two of them are threat and the identity processes involved with migration. Regarding threat, it is proposed that this may occur due to the competition generated in the labor market by the arrival of people and potential changes in wages or the availability of jobs resulting from their presence. Attitudes toward threat can vary significantly according to social position as migrants tend to take jobs that require lower skills and/or qualifications. In that sense, unskilled non-migrant workers can compete for the same jobs (Givens, 2007; Orrenius and Zavodny, 2009). A second explanation refers to the fiscal impact and the competition for benefits and social services that migration may generate. Once again, social position conditions this competition for access to health, education, or other relevant social assistance (Jaime-Castillo et al., 2016). The threat manifests itself in different ways, mainly as negative feelings or emotions in the interaction or the development of certain stereotypes about migrants (Croucher, 2017).

The notion of identity in the context of migration refers to the perception of moral differences (or similarities), values, customs, beliefs, or cultural practices (Stephan et al., 2000), considered central aspects of identity construction according to psychological perspectives. This notion has been part of the debate in migration and diaspora studies. As Hall (1990) argues, no identity exists without relations of difference, so the multicultural encounter that migration brings allows individuals' identity formation. Identity is not only a private psychological process but also a public matter, as it molds a “shared and communal sense of belonging with others and against Others” (Georgiou, 2006, p. 45). The notion that Benedict Anderson (2006) has of the nation, as a political “imagined community” and what Balibar (1991) calls a “fictive ethnicity”—which refers to the lack of ethnic basis of any nation-state—is key for understanding this sense of identity and the perceived threat represented by growing migration. According to Anderson (2013, p. 2), any modern state portrays itself as a “community of value”, whereby people share (non-arbitrary) values and patterns of behavior expressed by their culture, ethnicity (although fictitious), religion, and/or language. Valued as such, the community of “good citizens” requires protection from “outsiders” (2013, p. 3). As Goldberg (2001, p. 16) suggests, the state articulates itself nationally as racial and culturally homogeneous in order to create and maintain a unified national community. In that sense, the emergence of migratory movements and the production of heterogeneous societies have challenged nation-states, and the perception of migration as a threat usually elicits feelings of national identity (Goldberg, 2001).

### Empirical approaches to the study of attitudes toward migration

The study of the migratory phenomenon and the understanding of how people perceive it and behave accordingly has been approached from multiple methodological and disciplinary perspectives. From a qualitative approach, a vast production of studies emphasizes how perceptions about the migration phenomenon are constructed (see Zapata-Barrero and Yalaz, 2022). In contrast, despite the growing availability of comparative studies of public opinion with some focus on migration (i.e., ESS, ISSP, and WVS), the use of survey-quantitative data for studying attitudes toward migration is still less common than the qualitative approach, let alone the use of panel-type data even in Global North countries [Salamońska, 2022; see Eisnecker's (2019) analysis based on a longitudinal study in Germany].

Regarding the study of attitudes toward groups of migrants in survey research, it is possible to distinguish between the focus on negative or positive attitudes. Negative attitudes toward migrants deal with concepts such as prejudice, attitudes toward ethnic minorities, xenophobia, and threat or discrimination toward particular groups. As far as the study of positive attitudes is concerned, it can be traced back to research that evaluates people's opinions about developing a friendship or expressing positive feelings toward others (Bergamaschi and Santagati, 2019; Baldner et al., 2020). It is possible to link these types of studies with the idea of friendly coexistence as it captures the extent to which people are more willing to coexist with others who are perceived as different from them. In this sense, aspects such as the development of an intergroup friendship or positive emotions in coexistence can be considered as feeling “at ease” in the interaction. Another important source of the study of positive attitudes comes from research that evaluates support for multiculturalism or the willingness to support the maintenance of identities or cultural practices of others (Berry, 2001; Goodman and Alarian, 2021). Here, attitudes linked to intergroup identity are evaluated to the extent that they capture the willingness of non-migrants to live with others who maintain their cultural characteristics as long as they do not threaten local identity.

Measuring positive and negative attitudes toward migration offers a wide variety of concepts and measurement instruments in quantitative studies. First, the general study of the opposition to migration seeks to understand the opinion of non-migrant citizens about more closed or open migration policies or to receive migrants. It is typically evaluated in representative opinion surveys using a general question or a set of indicators treated as a composite index. For instance, the World Values Survey assesses opposition by using a series of questions to measure people's willingness to accept people from low-income countries or other ethnicities into “their” countries, prejudice toward migrants, perceived threat, support for maintenance of cultural practices or positive emotions, such as sympathy, trust or lack of anxiety (Meuleman et al., 2009). Second, prejudice is commonly assessed using multi-item scales that measure people's disposition toward particular groups of migrants. However, it is possible to find studies using prejudice measurements as opposed to migration (Pettigrew et al., 2007), sometimes used as interchangeable indicators. Third, the perception of threat addresses the effect that competition would have on certain resources or the distribution of goods that may be perceived as threatened in migratory contexts. Specifically, the measurements aim to assess to what extent non-migrants perceive that the arrival of migrants can impact educational provision, the labor market, or threaten national identity. Although it is used as an antecedent of the development of attitudes such as prejudice, it is also used as a dependent variable (Meuleman et al., 2009; Davidov et al., 2018). Finally, positive attitudes are evaluated using multiple items to measure people's willingness to support the maintenance of cultural practices, the degree of identity similarity, or the positive emotions that interaction with others can generate, such as sympathy, trust, or lack of anxiety. In all cases, the concepts are measured using items answered on a Likert-type scale that allows measuring the disposition of people to each concept.

## Factors associated with attitudes toward migration

Regarding the antecedents that have been used in the literature to explain the attitudinal differences, it is possible to classify them into individual and contextual theories of the development of attitudes toward migrants and migration (Quillian, 1995; Ceobanu and Escandell, 2010). On the one hand, at the individual level, two of the most relevant theories refer to socioeconomic resources and levels of intergroup contact. In terms of resources, multiple studies consistently show that people with lower educational levels or in lower social positions tend to develop more unfavorable attitudes toward migrants. This would also be particularly relevant in critical economic conditions (Ceobanu and Escandell, 2010; Meuleman et al., 2020; Bonhomme, 2021, 2022), while people with more resources tend to support greater equality of rights or positive attitudes toward migration (Miranda et al., 2018). The explanations for the effect of resources, particularly education, can be understood from the perspective of competence or enlightenment. The “labor market competition model” or “threat to status model” (Côté and Erickson, 2009; Jaime-Castillo et al., 2016) suggests that competition for scarce resources can vary depending on the social position of people. In lower socioeconomic levels, there is a tendency for more hostile attitudes given the greater competition for job or educational opportunities, which conditions the development of attitudes (Kunovich, 2004; Caro and Schulz, 2012). Furthermore, the evidence suggests that more educated people internalize democratic norms and principles to a greater extent (Lipset, 1960; Jackman and Muha, 1984), leading to a more positive attitudinal development.

A complementary alternative explanation to attitudes toward migrants comes from contact theory. This theory suggests that intergroup contact, from mere knowledge to the development of friendships, would allow non-migrants to establish daily relationships with migrants. The evidence tends to support that people who develop higher levels of contact with migrants—especially the best forms of contact, such as friendship—would improve their attitudes (by lessening the prejudice and perception of threat) toward them (Tropp and Pettigrew, 2005; Pettigrew and Tropp, 2008; Paluck et al., 2019).

Finally, at the contextual level, the focus has mostly been on structural socioeconomic conditions. For example, extending the concept of threat to a contextual level, it is argued that migration would generate intergroup competition for available resources (Ceobanu and Escandell, 2010; Jaime-Castillo et al., 2016). Therefore, a higher rate of migrants in a particular territory could condition attitudinal development, an impact that would increase in contexts with a growing migration rate, as is the Chilean case.

Following the previous literature, it is possible to propose the following hypotheses:

**H1**: non-migrants would show an increase in negative attitudes toward migrants over time (in terms of conviviality, identity, and perception of threat), particularly after the outbreak of COVID-19.
**H2:** the increase in negative attitudes toward migrants (in terms of conviviality, identity, and perception of threat) would be stronger for those with lower status.
**H3**: the increase in negative attitudes toward migrants (in terms of conviviality, identity, and perception of threat) would be stronger for those living in territories with a high rate of migrant residents.
**H4**: non-migrants with lower status and more interaction with migrants would increase their negative attitudes toward migration over time (in terms of conviviality, identity, and perception of threat).

The pre-registration of the hypothesis of the study can be found in the following link: https://osf.io/2npuq/?view_only=fe51f22a4d2340c1a0463d0ebca4b076.

## Data, variables, and methods

### Data

The main data source is the Chilean Longitudinal Social Survey (ELSOC) 2016–2021. ELSOC has been designed to evaluate yearly the way in which individuals think, feel and behave regarding a set of social issues related to conflict and social cohesion in Chile. The sampling design is probabilistic, stratified, clustered, and multistage. It provides adequate coverage of the country's largest cities (Metropolitan Area of Santiago, Valparaíso, and Concepción) and smaller cities comprising a total of 2,927 participants aged between 18 and 75 years on wave 1. It is representative of people in the north and south of the country. In addition, the sample has representativeness of 77% of the country's total population and 93% of the urban population, with a response rate of 62.4% (Centre for Social Conflict and Cohesion Studies, 2022).

The survey has been conducted yearly since 2016, with the exception of the year 2020, when it was suspended due to the pandemic. The administration of the questionnaire is face-to-face, but in the last wave (2021), it was conducted entirely over the phone. In 2018, wave 3 included a refreshment sample in order to counter survey attrition. The same sampling strategy of wave 1 was implemented for selecting the new cases. As a result, the total sample of wave 3 included 3,748 cases, of which 2,229 are part of the original sample, and 1,519 are from the refreshment sample. The data from the refreshment sample is not included in this article because we wanted to analyze a longer trend, thus, only cases from the original sample are employed in the analytical sample. Regarding the original sample, the response rate was 62.4% in wave 1, achieving *N* = 2,927 participants. The attrition in subsequent waves was 15.5% in wave 2 (*N* = 2,473), 9.9% in wave 3 (*N* = 2,229), 3.4% in wave 4 (*N* = 2,153), and 19.2% in wave 5 (*N* = 1,739). In broader terms, the accumulated attrition between wave 1 and wave 5 is 40.5%. A limitation of this study is that sampling weights unfortunately were not available in the dataset for longitudinal analysis. For a more detailed analysis of responses and attrition, visit https://coes.cl/encuesta-panel/.

Regarding the questions about migrants, the first three waves referred only to Peruvians, and from wave 4th (2019) onwards, the sample was split: one half included questions about Peruvians and the other half about Venezuelans, as they both became one of the largest migratory groups in Chile. For the analysis, both groups are combined in one general category of “migrants”, but there will be a dummy variable controlling for this difference in the models (Venezuelans = 1, Peruvians = 0). The detail for each wave is depicted in Table 1.

**Table 1:** Summary of the original sample.

**Target migratory group**	**Wave**					**Total**
	**2016**	**2017**	**2018**	**2019**	**2021**	
Peruvians	2.927	2.473	2.229	1.100	846	9.575
Venezuelans	0	0	0	1.053	893	1.946
**Total**	2.927	2.473	2.229	2.153	1.739	11.521
**Data procedures**						
1. Fix to wave 5	1.739	1.739	1.739	1.739	1.739	8.695
2. Listwise deletion	1.173	1.208	1.261	1.286	1.416	6.344
Missing (%)	32.55	30.53	27.49	26.05	18.57	27.04

Target migratory group means that the questions on attitudes toward migration are asked specifically about these groups. For example, in waves 1–5, the statement says, my family values that I have “Peruvians” friends. From 2019 onwards, a part of the sample was asked for “Venezuelans” instead of “Peruvians”.

Table 1 summarizes each wave's total number of cases and the data processing rationale. First, the sample is fixed to the number of cases present on the last wave (*N* = 1,739). Second, we applied a listwise deletion that keeps all the cases with complete information in the variables of interest. Finally, after missing data cleaning, the final dataset comprises 1,611 individuals, corresponding to 6,344 observations over the five waves nested within 93 municipalities. No data imputation methods were used in the final analytical sample.

For the contextual data at the municipality level, we use data from the National Socio-Economic Characterization Survey (CASEN) for the years 2017 (*N* = 216,439) and 2020 (*N* = 185,437) (Ministerio de Desarrolllo Social y Familia, 2017, 2021). CASEN is a national probabilistic, stratified, two-stage household survey representative of the overall urban and rural population of Chile with 18 years of age or older achieving a response rate of 75.5%. In 2017 the survey was conducted using face-to-face CAPI interviews with the head of household. Because of the pandemic, in 2020, the survey switched from single-mode face-to-face to mixed telephone mode with limited face-to-face interviews. Nevertheless, the sampling design remained stable, achieving a response rate of 63.1%. The computation for the variables at the municipality level is described in the next section. The data is available at: http://observatorio.ministeriodesarrollosocial.gob.cl/encuesta-casen. The last procedure was merging the individual-level panel data with the CASEN survey information for the 93 municipalities using the unique administrative identification number available in both datasets. No missing data were reported regarding the variables of interest at the municipality level.

### Variables

The main dependent measures refer to three aspects of social cohesion related to non-migrants' attitudes toward migrants: *Convivial/Conviviality, Identity*, and *Threat*. The first variable corresponds to the average of five statements measured by Likert scales that captures the extent to which people agree with different aspects of conviviality (α = 0.75). The second variable is the average of four Likert scales that seek to capture non-migrants' attitudes toward migrants regarding national identity and costumes (α = 0.54). Finally, we use a single-item question to capture the agreement with the idea that migrants constitute a threat in terms of the increase in unemployment. For details of each item, see Table 2.

**Table 2:** Items for perceptions and attitudes toward migrants.

**Concept**	**ID**	**Item**	**Question**	**Categories**
Conviviality	c01	Interaction anxiety with (PER/VEN)	If you had to talk with a group of (Peruvians/Venezuelans) who live in Chile and that you don't know, how would you feel?	1. Very uncomfortable
				5. Very comfortable
	c02	Sympathy for (PER/VEN) living in Chile	How much do you like the (Peruvians/Venezuelans) living in Chile?	1. Very little or not at all
				5. A lot
	c03	Family value of migrant friends (PER/VEN)	My family values that I have (Peruvians/Venezuelans) friends.	1. Strongly agree
				5. Strongly disagree
	c04	Friends value of migrant friends (PER/VEN)	My friends value that I have (Peruvians/Venezuelans) friends.	1. Strongly agree
				5. Strongly disagree
	c05	Migrants (PER/VEN) have Chilean friends	How much do you agree or disagree with that (Peruvians/Venezuelans) living in Chile have Chilean friends?	1. Strongly agree
				5. Strongly disagree
Identity	i01	Similarity between Chileans and (PER//VEN)	How similar among them are Chileans and (Peruvians/Venezuelans) living in Chile?	1. Not similar
				5. Very similar
	i02	Loose of identity because of migrants	With the arrival of so many (Peruvians/Venezuelans), Chile is losing its identity.	1. Strongly agree
				5. Strongly disagree
	i03	Migrant keeping their customs	How much do you agree or disagree with (Peruvians/Venezuelans) living in Chile keeping their customs and traditions?	1. Strongly agree
				5. Strongly disagree
	i04	Adoption of Chilean customs	How much do you agree or disagree with (Peruvians/Venezuelans) living in Chile adopting Chilean customs and traditions?	1. Strongly agree
				5. Strongly disagree
Threat	t01	Unemployment increases	With the arrival of so many (Peruvians/Venezuelans) Chile is increasing unemployment.	1. Strongly agree
				5. Strongly disagree

For measuring social status, we use educational level, household income quintiles, and subjective social status. In order to better reflect the attitudes of lower-status individuals in the models, we set the highest educational level and income quintile as reference categories. Regarding subjective social status, we use a reverse coded measure, in which each increase in the scale represents a lower individual status perception.

To capture the influence of interaction and friendship with migrants on social cohesion attitudes, we use two variables that have been measured in wave 1 (2016) and wave 4 (2019): *number of known migrants* and *number of migrant friends*. The dummy coded variables were 0 = no known/friends and 1 = one or more. The changes between waves are coded in four groups: (1) Stable, do not know/do not have friends; (2) Stable, know/have friends; (3) Now know/have friends, and; (4) No longer know/have friends. The control variables gender, age, and nationality are included in the estimations.

The descriptives of the individual-level variables are presented in Table 3.

**Table 3:** Descriptive statistics of individual data.

**Variable**	**Stats/values**	**Freqs (% of valid)**	**Graph**
Convivial/Conviviality	Mean (sd): 3.4 (0.6)	21 distinct values	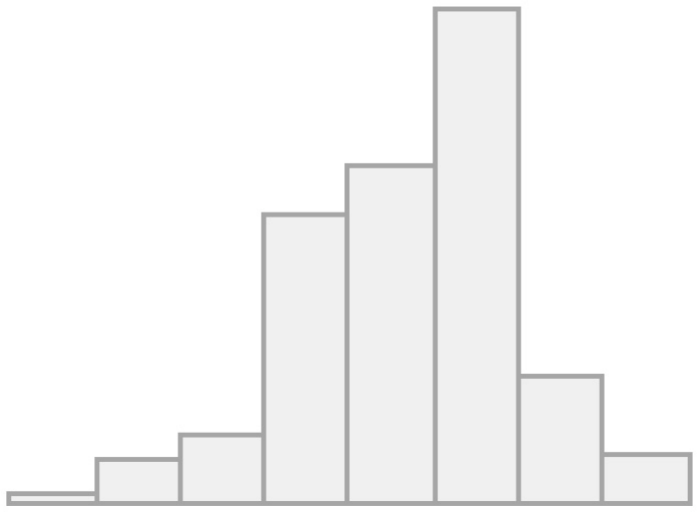
	min ≤ med ≤ max: 1 ≤ 3.4 ≤ 5		
Identity	Mean (sd): 3.3 (0.7)	17 distinct values	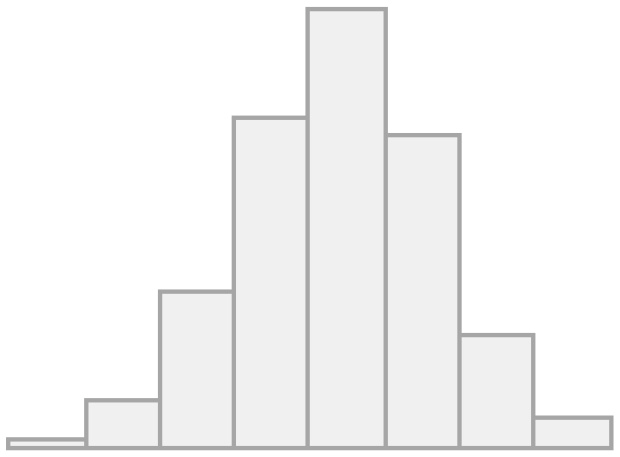
	min ≤ med ≤ max: 1 ≤ 3.2 ≤ 5		
Threat (unemployment increases)	1. Strongly disagree	447 (7.0% )	
	2. Disagree	1,889 (29.8%)	
	3. Neither disagree nor agree	760 (12.0%)	
	4. Agree	2,504 (39.5%)	
	5. Strongly agree	744 (11.7%)	
Education	1. Universitary	1,262 (19.9%)	
	2. Technical	1,069 (16.9%)	
	3. High school	2,769 (43.6%)	
	4. Primary	1,244 (19.6%)	
Household income quintile per capita (NA)	1. Q5	1,174 (18.5%)	
	2. Q4	1,193 (18.8%)	
	3. Q3	1,258 (19.8%)	
	4. Q2	1,221 (19.2%)	
	5. Q1	1,226 (19.3%)	
	6. QNA	272 (4.3% )	
Subjective social status: individual (reverse)	Mean (sd): 5.6 (1.5)	11 distinct values	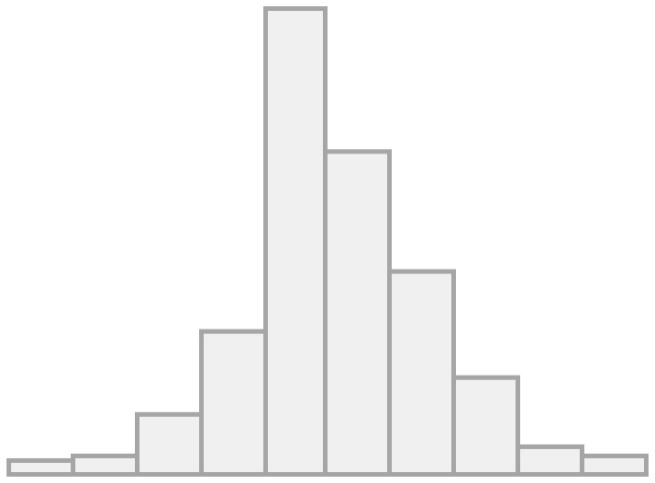
	min ≤ med ≤ max: 0 ≤ 5 ≤ 10		
Know migrants (diff. t4–t1)	1. Stable, do not know	3,337 (52.6%)	
	2. Stable, know	905 (14.3%)	
	3. Now know	871 (13.7%)	
	4. No longer know	1,231 (19.4%)	
Have migrant friends (diff. t4–t1)	1. Stable, do not have friends	4,201 (66.2%)	
	2. Stable, have friends	523 (8.2%)	
	3. Now have friends	735 (11.6%)	
	4. No longer have friends	885 (14.0%)	
Age groups	1.18–29	1,023 (16.1%)	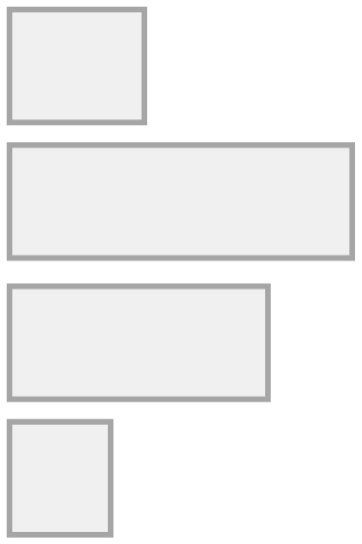
	2.30–49	2,586 (40.8%)	
	3.50–64	1,956 (30.8%)	
	4.65 or more	779 (12.3%)	
Gender	1: Male	2,334 (36.8%)	
	2: Female	4,010 (63.2%)	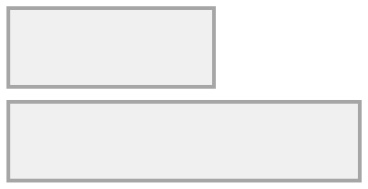

Own elaboration based on ELSOC Survey. *N* = 6,334 (all waves).

The variables for the municipality level are shown in Table 4. For the variable *Percentage of migrants at the municipality level*, we use the question about the country where the mother of the respondent was living at the moment of his/her/their birth; the computation is based on the proportion of individuals that declare to be born outside Chile over the total population of the municipality using populations weights at this administrative level that are provided by the data.

**Table 4:** Descriptive statistics of municipality data.

**Variable**	**Stats/values**	**Graph**
Proportion of migrant population (weighted)–CASEN 2017–Municipality	Mean (sd): 2.2 (5.1)	
	min ≤ med ≤ max: 0 ≤ 1.1 ≤ 42.4	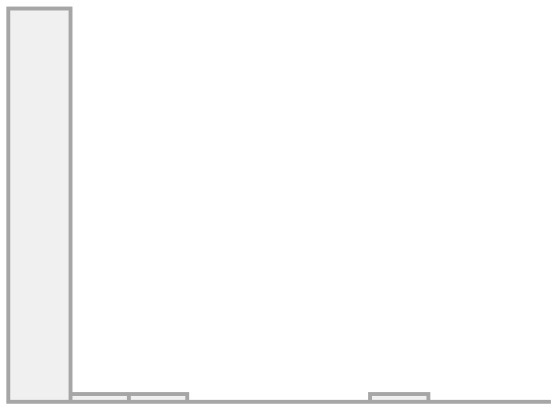
Proportion of migrant population (weighted)—CASEN 2020—Municipality	Mean (sd): 3.6 (5.2)	
	min ≤ med ≤ max: 0 ≤ 2.1 ≤ 35.3	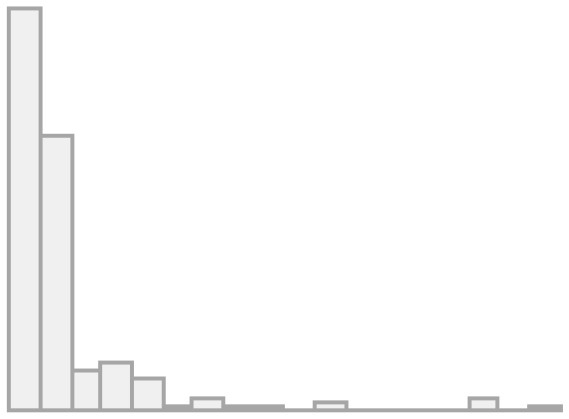
Change in the proportion of the migrant population between 2017 and 2020—CASEN	Mean (sd): 1.3 (2.1)	
	min ≤ med ≤ max: −7.1 ≤ 0.9 ≤ 14	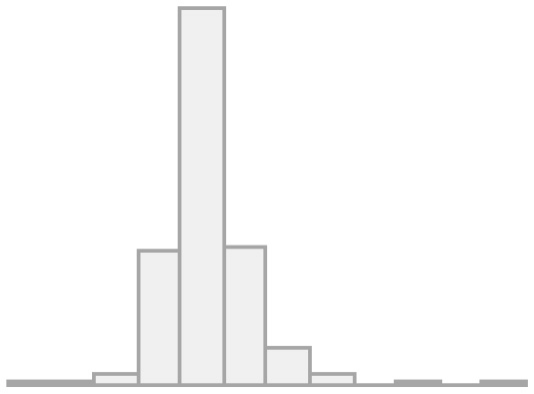

Own elaboration based on CASEN Survey. *N* = 93 (municipalities).

For the computation of this measurement, we considered the large two migratory groups in Chile in the last 5 years: Venezuela (30.7%), and Peru (16.3%). Second, the variable *Change in the percentage of migrants at the municipality level* aims to measure the temporal changes within the municipality. Therefore, the variable was computed as the difference between the percentage of migrants between 2017 and 2020.

### Methods

Given the hierarchical structure of the data (observations nested in surveys nested in municipalities), we applied a longitudinal multilevel strategy (Singer and Willett, 2003). Longitudinal multilevel models are suited to account for the shared variance among units in the data for better estimation of standard errors. Given that individuals over time share variance within themselves, if the error structure is not taken into account, then it would be as if they were considered different individuals. Multilevel models allow a solution in regression estimation by adding a random term that represents the variance associated with the nesting of the data (random effects). The linear multilevel models are estimated using the R library “lme4” (Bates et al., 2015, p. 4).

The estimated multilevel model can be formalized as follows:


$$\begin{array}{l} y_{tjk}=\gamma_{000}+Wave_{jk}+Status_{jk}+Know_{jk}+Friend_{jk}+\\ PropMig_{k}+ChangeMig_{k}+\mu_{00k}+\;r_{0jk}+e_{tjk} \end{array}$$

Where,

- *y*_*tjk*_: is the value of the repeated measures on attitudes toward migration.
- *Wave*: is the measurement of time.
- *Status*: is the socioeconomic status of the individual.
- *Know*: indicates if the respondent knows (or knew) at least one migrant.
- *Friend*: indicates if the respondent has (or had) at least one migrant friend.
- *PropMig*: is the proportion of the migrant population at the municipality level.
- *ChangeMig*: is the change, between 2017 and 2020, in the proportion of the migrant population at the municipality level.
- μ_00*k*_, *r*_0*jk*_, and *e*_*tjk*_ are the error terms at the municipality, individual, and observation levels, respectively.

And adding interactions with time (wave) for assessing longitudinal changes:


$$\begin{array}{l} y_{tjk}=\gamma_{000}+Wave_{jk}+Status_{jk}\times Wave_{jk}+\\ Know_{jk}\times Wave_{jk}+Friend_{jk}\times Wave_{jk}+\\ ChangeMg_{k}\times Wave_{jk}+\\ \mu_{00k}+\;r_{0jk}\times Wave_{jk}+e_{tjk} \end{array}$$

Where,

- *Status*_*jk*_ × *Wave*_*jk*_ is the interaction effect of time with *Status* (the same as with *Know, Friend*, and *ChangeMig*)
- *r*_0*jk*_ × *Wave*_*jk*_ is the random slope variation for the slope of *Wave*.

## Results

### Descriptives

**Figure 1:**
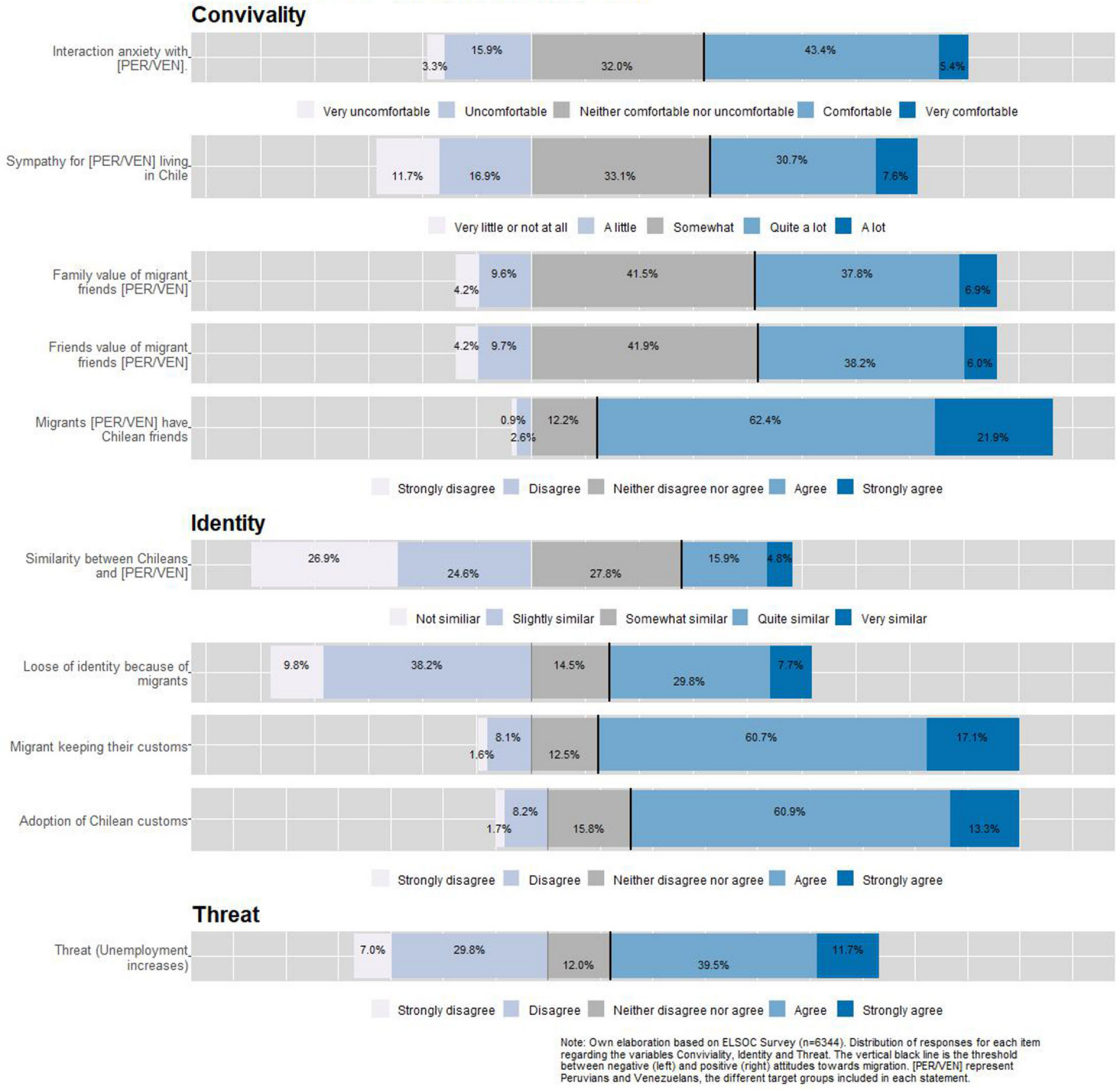
Frequencies for convivial/conviviality, identity, and threat items.

Figure 1 shows the univariate descriptives for the items that will be later used in the multilevel regression models as indexed dependent variables. For the items of the conviviality dimension, we observe that almost half feel comfortable in their interaction with migrants, and more than a third feel sympathy for them. Less than half of the respondents' friends and family value friendship with migrants, whereas a great majority agree that migrants can have Chilean friends, meaning this is seen as something positive. This is interesting as it seems more valuable that LAC migrants have Chilean friends than Chileans people having LAC migrant friends. Regarding the items on the identity dimension, a minority (20%) find similarities between migrants and non-migrants, pointing to a large recognition of differences. Almost 40% are concerned about the potential identity loss due to migration, whereas more than two-thirds agrees on the relevance of maintaining their own culture as well as incorporating the national one. Finally, half of the respondents show concerns about the impact of migration on unemployment.

**Figure 2:**
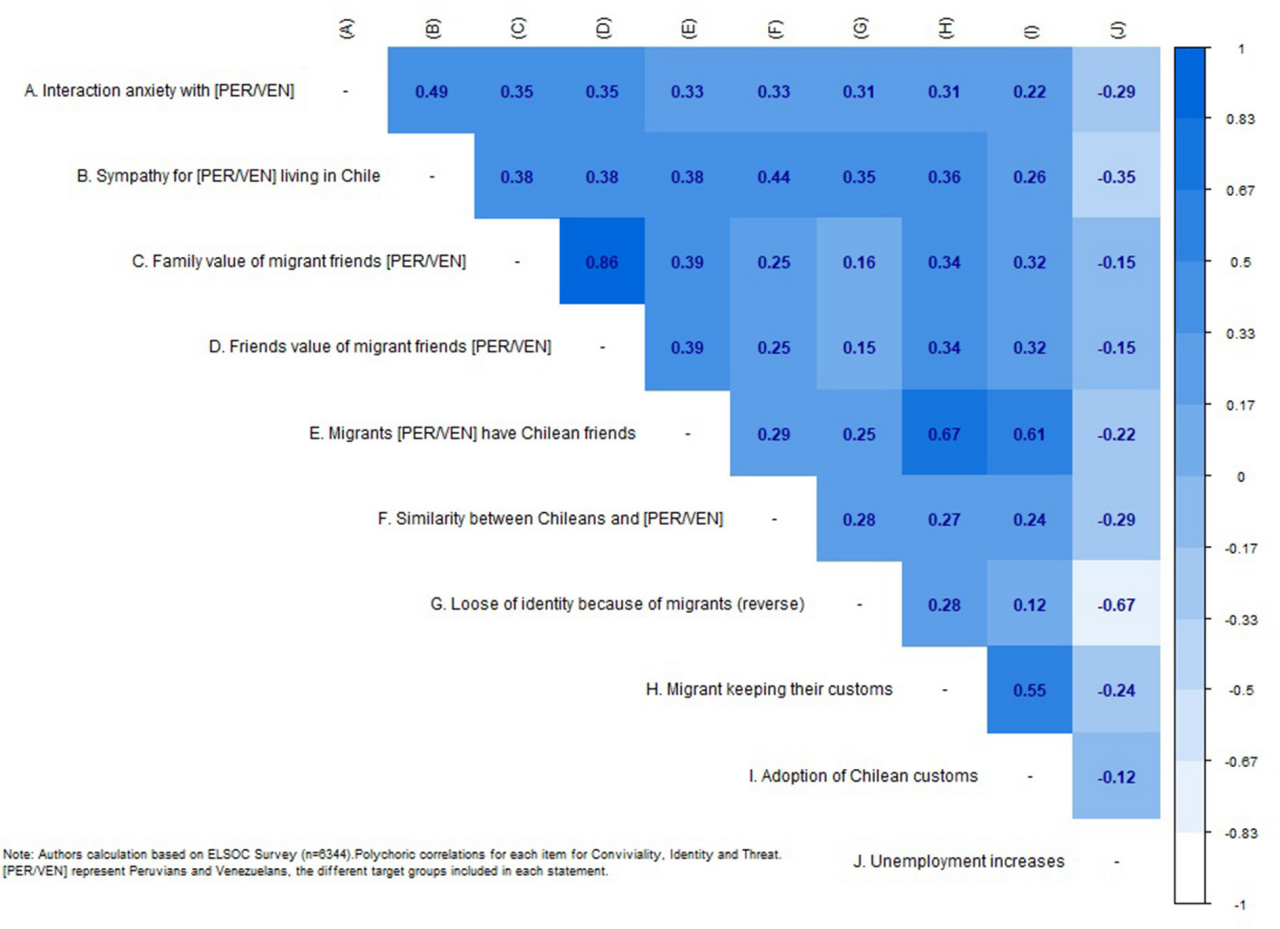
Polychoric correlation matrix for convivial/conviviality, identity, and threat items.

First, we will analyze the correlation matrix of the items that make up the different dimensions of the dependent variable, which are presented in Figure 2, using the data from all survey waves.

As we can see, the correlations generally have values between moderate and high, which indicates a certain level of association between the indicators and dimensions. The correlations in the dimension of conviviality (items A–E) move in the range between 0.33 and 0.86, while those of identity (F–I) have somewhat lower values (0.12–0.55). The perception of threat (J), which represents the third dimension, has a negative relationship with all the indicators. This was expected as the other variables are coded in the positive sense of social cohesion.

### Multilevel regression models

In the following we present the results of the estimation of the multilevel regression models for each of our three dependent variables, beginning with Table 5 which shows the results of the estimation for conviviality. The first variable in Model 1 is wave, which depicts a general perspective of the variations of the dependent variable over time. We observe that in relation to the reference category (wave 1) conviviality is significantly higher in waves 2, 3, and 4, but then it becomes even negative in the last wave (2021). This result is noteworthy as the last wave was carried out in times of the coronavirus pandemic.

**Table 5:** Multilevel linear regression models for conviviality.

	**Model 1**	**Model 2**	**Model 3**	**Model 4**
**Wave (ref: wave 2016)**				
Wave 2017	0.07 (0.02)^**^	0.07 (0.02)^**^	0.07 (0.02)^***^	0.07 (0.02)^***^
Wave 2018	0.11 (0.02)^***^	0.11 (0.02)^***^	0.11 (0.02)^***^	0.11 (0.02)^***^
Wave 2019	0.07 (0.02)^**^	0.07 (0.02)^**^	0.07 (0.02)^**^	0.07 (0.02)^**^
Wave 2020	−0.05 (0.02)^*^	−0.05 (0.02)^*^	−0.05 (0.02)^*^	−0.05 (0.02)^*^
**Education (ref: Universitary)**				
Technical		−0.16 (0.04)^***^	−0.15 (0.04)^***^	−0.15 (0.04)^***^
High school		−0.26 (0.03)^***^	−0.24 (0.03)^***^	−0.24 (0.03)^***^
Primary		−0.39 (0.04)^***^	−0.38 (0.04)^***^	−0.38 (0.04)^***^
**Household income (ref: quintile 5)**				
Quintile 4		−0.09 (0.04)^*^	−0.08 (0.04)^*^	−0.08 (0.04)^*^
Quintile 3		−0.08 (0.04)^*^	−0.08 (0.04)^*^	−0.08 (0.04)^*^
Quintile 2		−0.10 (0.04)^*^	−0.09 (0.04)^*^	−0.09 (0.04)^*^
Quintile 1		−0.12 (0.04)^**^	−0.10 (0.04)^*^	−0.11 (0.04)^**^
Subjective social status		−0.00 (0.01)	−0.00 (0.01)	−0.00 (0.01)
Woman (ref: man)		0.00 (0.02)	0.01 (0.02)	0.01 (0.02)
**Know migrant (ref: stable, do not know)**				
Stable, know			0.06 (0.04)	0.07 (0.04)
Now know			0.08 (0.04)^*^	0.09 (0.04)^*^
No longer know			0.04 (0.03)	0.06 (0.03)
**Migrant friend (ref: stable, do not have friends)**				
Stable, have friends			0.28 (0.05)^***^	0.29 (0.05)^***^
Now have friends			0.09 (0.04)^*^	0.08 (0.04)^*^
No longer have friends			0.09 (0.04)^*^	0.10 (0.04)^**^
**Municipality characteristics**				
Proportion of migrants				−0.01 (0.00)
Change in the proportion of migrants				−0.02 (0.01)^*^
AIC	11,338.60	11,260.69	11,235.70	11,245.00
BIC	11,473.71	11,463.35	11,478.88	11,501.70
Log likelihood	−5,649.30	−5,600.35	−5,581.85	−5,584.50
Likelihood-ratio test		152.3 (10)^***^	66.6 (6)^***^	13.3 (2)^**^
Num. obs.	6,344	6,344	6,344	6,344
L1: num. individual	1,611	1,611	1,611	1,611
L2: num. municipality	93	93	93	93
Var: individual (intercept)	0.14	0.13	0.12	0.12
Var: municipality (intercept)	0.01	0.00	0.01	0.00
Var: residual	0.26	0.26	0.26	0.26

*** *p* < 0.001;

** *p* < 0.01;

* *p* < 0.05.

Standard error in parenthesis. Model 1 shows the estimation for the average change between waves.

Models 2 y 3 show the estimations of the random intercept multilevel models of the individual social status and changes in contact and friendship with migrants.

The estimation considers the following nesting structure of the data: Observations, within Individuals which are also nested within Municipalities.

Model 2 incorporates socioeconomic status variables. Starting with education, we observe a negative association with conviviality (as university education is the reference category). In addition, the effect size increases as the educational level decreases, with those who reached primary education displaying the lowest level of convivial attitudes. Regarding the income quintiles, it is also possible to appreciate lower conviviality in the lower levels, although this result is weaker when compared to that of the educational level. The third status variable that is incorporated into the models is subjective social status, which does not show significant effects here and in any of the following models.

Model 3 presents variables covering relationships with migrants and their change over time, having as a reference category those who have not been related to migrants in all waves. In general, there are no consistent effects, although the only category that is positively related to greater conviviality is that of those who have increased their relationship with migrants over time. Regarding friendships, non-migrants who maintain friendships as well as those who decrease their friendships, show a higher level of conviviality. It could be concluded that those who at some point have been friends with migrants show greater conviviality (since the reference category is those who have never had migrant friends).

The contextual variables enter in Model 4, where we can observe that the net presence of migrants at the commune level does not have an effect on conviviality, but its increase over time does, leading to a decrease in conviviality. Even though the effect is small, it is consistent across models.

Table 6 shows the results for the models on our second dependent variable: identity. Model 1 shows the effect of time, with an increase in the first waves and then a decrease in the last, a similar pattern to what happened with convivial/conviviality but with a non-significant decrease in the 2020's wave. Consistent with the models for conviviality, in Model 2 the groups with the lowest educational level show the most negative attitudes. Regarding relationships with migrants, Model 3 shows that those who have increased their knowledge of migrants have more positive attitudes in the identity dimension, as do those who have stable friendship relationships. The contextual variables (Model 4) in this case do not render significant effects.

**Table 6:** Multilevel linear regression models for identity.

	**Model 1**	**Model 2**	**Model 3**	**Model 4**
**Wave (ref: wave 2016)**				
Wave 2017	0.10 (0.02)^***^	0.10 (0.02)^***^	0.10 (0.02)^***^	0.10 (0.02)^***^
Wave 2018	0.11 (0.02)^***^	0.11 (0.02)^***^	0.11 (0.02)^***^	0.11 (0.02)^***^
Wave 2019	0.15 (0.02)^***^	0.15 (0.02)^***^	0.15 (0.02)^***^	0.15 (0.02)^***^
Wave 2020	0.01 (0.02)	0.01 (0.02)	0.02 (0.02)	0.01 (0.02)
**Education (ref: Universitary)**				
Technical		−0.20 (0.04)^***^	−0.19 (0.04)^***^	−0.19 (0.04)^***^
High school		−0.37 (0.03)^***^	−0.36 (0.03)^***^	−0.36 (0.03)^***^
Primary		−0.50 (0.04)^***^	−0.50 (0.04)^***^	−0.50 (0.04)^***^
**Household income (ref: quintile 5)**				
Quintile 4		−0.09 (0.04)^*^	−0.09 (0.04)^*^	−0.09 (0.04)^*^
Quintile 3		−0.06 (0.04)	−0.06 (0.04)	−0.06 (0.04)
Quintile 2		−0.06 (0.04)	−0.06 (0.04)	−0.06 (0.04)
Quintile 1		−0.08 (0.04)	−0.07 (0.04)	−0.07 (0.04)
Subjective social status		−0.01 (0.01)	−0.01 (0.01)	−0.01 (0.01)
Woman (ref: man)		−0.05 (0.02)^*^	−0.05 (0.02)^*^	−0.05 (0.02)^*^
**Know migrant (ref: stable, do not know)**				
Stable, know			0.05 (0.04)	0.06 (0.04)
Now know			0.07 (0.04)^*^	0.08 (0.04)^*^
No longer know			0.01 (0.03)	0.01 (0.03)
**Migrant friend (ref: stable, do not have friends)**				
Stable, have friends			0.19 (0.05)^***^	0.19 (0.05)^***^
Now have friends			−0.01 (0.04)	−0.01 (0.04)
No longer have friends			−0.01 (0.04)	−0.01 (0.04)
**Municipality characteristics**				
Proportion of migrants				−0.01 (0.00)
Change in the proportion of migrants				−0.01 (0.01)
AIC	11,498.72	11,332.58	11,341.71	11,358.37
BIC	11,633.82	11,535.23	11,584.90	11,615.07
Log likelihood	−5,729.36	−5,636.29	−5,634.86	−5,641.19
Likelihood-ratio test		241.5 (10)^***^	32.1 (6)^***^	5.3 (2)
Num. obs.	6,344	6,344	6,344	6,344
L1: num. individual	1,611	1,611	1,611	1,611
L2: num. municipality	93	93	93	93
Var: individual (intercept)	0.14	0.11	0.11	0.11
Var: municipality (intercept)	0.02	0.01	0.01	0.01
Var: residual	0.26	0.26	0.26	0.26

*** *p* < 0.001;

** *p* < 0.01;

* *p* < 0.05.

Standard error in parenthesis. Model 1 shows the estimation for the average change between waves.

Models 2 y 3 show the estimations of the random intercept multilevel models of the individual social status and changes in contact and friendship with migrants.

The estimation considers the following nesting structure of the data: Observations, within Individuals which are also nested within Municipalities.

Regarding our third dependent variable of perceived threats to employment due to the presence of migrants, Model 1 in Table 7 shows that it seems to be decreasing over time. Consistent with previous models, those with a lower educational level are more threatened by unemployment (Model 2), contrary to those who have stable migrant friends (Model 3). As in the case of the identity variable, there are no effects at the contextual level.

**Table 7:** Multilevel linear regression models for threat.

	**Model 1**	**Model 2**	**Model 3**	**Model 4**
**Wave (ref: wave 2016)**				
Wave 2017	−0.17 (0.04)^***^	−0.17 (0.04)^***^	−0.17 (0.04)^***^	−0.17 (0.04)^***^
Wave 2018	−0.19 (0.04)^***^	−0.19 (0.04)^***^	−0.19 (0.04)^***^	−0.19 (0.04)^***^
Wave 2019	−0.16 (0.04)^***^	−0.16 (0.04)^***^	−0.16 (0.04)^***^	−0.16 (0.04)^***^
Wave 2020	−0.11 (0.04)^*^	−0.11 (0.04)^**^	−0.11 (0.04)^**^	−0.11 (0.04)^**^
**Education (ref: Universitary)**				
Technical		0.37 (0.07)^***^	0.36 (0.07)^***^	0.36 (0.07)^***^
High school		0.66 (0.06)^***^	0.65 (0.06)^***^	0.65 (0.06)^***^
Primary		0.83 (0.07)^***^	0.82 (0.07)^***^	0.82 (0.07)^***^
**Household income (ref: quintile 5)**				
Quintile 4		0.09 (0.07)	0.08 (0.07)	0.09 (0.07)
Quintile 3		0.02 (0.07)	0.02 (0.07)	0.03 (0.07)
Quintile 2		0.05 (0.07)	0.04 (0.07)	0.05 (0.07)
Quintile 1		0.08 (0.07)	0.06 (0.07)	0.07 (0.07)
Subjective social status		0.00 (0.01)	0.00 (0.01)	0.00 (0.01)
Woman (ref: man)		0.04 (0.04)	0.04 (0.04)	0.04 (0.04)
**Know migrant (ref: stable, do not know)**				
Stable, know			−0.02 (0.08)	−0.04 (0.08)
Now know			−0.02 (0.07)	−0.03 (0.07)
No longer know			−0.04 (0.06)	−0.05 (0.06)
**Migrant friend (ref: stable, do not have friends)**				
Stable, have friends			−0.27 (0.09)^**^	−0.28 (0.09)^**^
Now have friends			−0.13 (0.07)	−0.13 (0.07)
No longer have friends			0.02 (0.07)	0.02 (0.07)
**Municipality characteristics**				
Proportion of migrants				0.01 (0.00)
Change in the proportion of migrants				0.00 (0.01)
AIC	19,128.30	19,000.78	19,017.57	19,035.33
BIC	19,263.41	19,203.44	19,260.76	19,292.03
Log likelihood	−9,544.15	−9,470.39	−9,472.79	−9,479.67
Likelihood-ratio test		190.7(10)^***^	17.1(6)^**^	2.3(2)
Num. obs.	6,344	6,344	6,344	6,344
L1: num. individual	1,611	1,611	1,611	1,611
L2: num. municipality	93	93	93	93
Var: individual (intercept)	0.45	0.38	0.38	0.38
Var: municipality (intercept)	0.02	0.01	0.01	0.01
Var: residual	0.89	0.89	0.89	0.89

*** *p* < 0.001;

** *p* < 0.01;

* *p* < 0.05.

Standard error in parenthesis. Model 1 shows the estimation for the average change between waves.

Models 2 y 3 show the estimations of the random intercept multilevel models of the individual social status and changes in contact and friendship with migrants.

The estimation considers the following nesting structure of the data: Observations, within Individuals which are also nested within Municipalities.

Finally, Table 8 shows the results of the interactions for the three dependent variables. These models attempt to explore to what extent some of the effects of previous models change significantly over time. Therefore, these interactions are part of the estimation with all the independent variables (Model 4 of the previous tables), but only the coefficients of the interactions are presented for the sake of space. In the model for conviviality (Model 1), in education, it is observed that it is the level of primary education—which maintained the most negative attitudes in the previous models—the one that would also show a decrease in conviviality over time. Regarding the relationships with migrants, those who increase their knowledge over time also increase in convivial attitudes, and the opposite happens for those who decrease their relationships with migrants, which is also replicated in the case of friendships. In the case of the identity variable (Model 2), the interactions show that attitudes on this realm become more negative over time for those with a lower educational level and for those whose knowledge of and/or friendship with migrants has decreased. Finally, the feeling of the threat of unemployment (Model 3) increases for those who have diminished their relationships with migrants over time.

**Table 8:** Multilevel linear regression models with interactions.

	**Model 1 (conviviality)**	**Model 2 (identity)**	**Model 3 (threat)**
Wave	0.00 (0.02)	0.02 (0.02)	−0.08 (0.03)^**^
Technical (ref: Universitary)	−0.14 (0.06)^*^	−0.13 (0.06)^*^	0.20 (0.11)
High school	−0.21 (0.05)^***^	−0.28 (0.06)^***^	0.46 (0.10)^***^
Primary	−0.18 (0.07)^**^	−0.32 (0.07)^***^	0.53 (0.12)^***^
Quintile 4 (ref: quintile 5)	−0.11 (0.06)	−0.17 (0.06)^**^	0.20 (0.11)
Quintile 3	−0.11 (0.06)	−0.16 (0.06)^*^	0.07 (0.11)
Quintile 2	−0.12 (0.07)	−0.11 (0.07)	0.15 (0.12)
Quintile 1	−0.12 (0.07)	−0.13 (0.07)	0.15 (0.12)
Stable, know (ref: stable, do not know)	0.09 (0.07)	0.06 (0.07)	−0.05 (0.12)
Now know	0.05 (0.06)	0.13 (0.06)^*^	−0.12 (0.12)
No longer know	0.14 (0.06)^**^	0.06 (0.06)	−0.21 (0.10)^*^
Stable, have friends (ref: stable, do not have friends)	0.24 (0.08)^**^	0.21 (0.08)^*^	−0.26 (0.15)
Now have friends	−0.11 (0.07)	−0.10 (0.07)	−0.12 (0.12)
No longer have friends	0.17 (0.06)^**^	0.07 (0.06)	−0.02 (0.11)
Change in the proportion of migrants	−0.02 (0.01)	−0.00 (0.01)	−0.02 (0.02)
**Education** **×wave**
Technical × wave	−0.00 (0.02)	−0.02 (0.02)	0.06 (0.03)
High school × wave	−0.01 (0.01)	−0.03 (0.01)	0.06 (0.03)^*^
Primary × wave	−0.06 (0.02)^***^	−0.06 (0.02)^***^	0.10 (0.03)^**^
**Quintile** **×wave**
Quintile 4 × wave	0.01 (0.02)	0.02 (0.02)	−0.04 (0.03)
Quintile 3 × wave	0.01 (0.02)	0.03 (0.02)^*^	−0.02 (0.03)
Quintile 2 × wave	0.01 (0.02)	0.02 (0.02)	−0.03 (0.03)
Quintile 1 × wave	0.00 (0.02)	0.02 (0.02)	−0.03 (0.03)
**Know migrant** × **wave**
Stable, know × wave	−0.00 (0.02)	0.00 (0.02)	0.00 (0.03)
Now know × wave	0.01 (0.02)	−0.02 (0.02)	0.03 (0.03)
No longer know × wave	−0.03 (0.02)^*^	−0.02 (0.01)	0.05 (0.03)^*^
**Migrant friend** × **wave**
Stable, have friends × wave	0.01 (0.02)	−0.00 (0.02)	−0.01 (0.04)
Now have friends × wave	0.06 (0.02)^***^	0.03 (0.02)	−0.00 (0.03)
No longer have friends × wave	−0.02 (0.02)	−0.03 (0.02)	0.01 (0.03)
**Migrant population** **×wave**
Change in the proportion of migrants × wave	−0.00 (0.00)	−0.00 (0.00)	0.01 (0.00)
AIC	11,315.15	11,491.05	19,123.35
BIC	11,666.43	11,842.33	19,474.63
Log likelihood	−5,605.58	−5,693.53	−9,509.68
Num. obs.	6,344	6,344	6,344
L1: num. individual	1,611	1,611	1,611
L2: num. municipality	93	93	93
Var: individual (intercept)	0.19	0.18	0.48
Var: individual wave	0.01	0.01	0.02
Cov: individual (intercept) wave	−0.03	−0.02	−0.04
Var: municipality (intercept)	0.00	0.01	0.01
Var: residual	0.23	0.25	0.86

*** *p* < 0.001;

** *p* < 0.01;

* *p* < 0.05.

Standard error in parenthesis. Model 1–3 shows the results of the interaction effects of time with socioeconomic status, know migrants, has migrant friend and change on migrant population. The time is specified as a random slope.

## Discussion

Chileans' attitudes toward migrants from Venezuela and Peru have significantly changed in the last years in different ways. The results show that, contrary to our first hypothesis, negative attitudes toward migrants actually decreased over time since 2016. Yet in early 2020 in Chile, this attitudinal improvement of non-migrants toward migrants was disrupted somehow, and convivial attitudes significantly decreased. At the same time, the perceptions of threat regarding Chileans' identity and customs and the potential job loss increased and returned to the previous levels of 2016. Thus, our predictions are only partially supported as the pandemic seems to have worked against the more positive trends we identified since 2016 regarding Chileans' perceptions toward Peruvian and Venezuelan migrants.

This study also shows that negative attitudes toward migrants are stronger in Chileans that have a lower status in society, in line with our second hypothesis. A key aspect to highlight is that, among the status variables, education shows a consistent effect in predicting the three aspects of social cohesion considered. Income nonetheless is only related to conviviality, while subjective social status is not related to any of the aspects of social cohesion. It seems that the objective dimensions of status, such as education, are more relevant than the subjective ones when it comes to explaining non-migrants' attitudes toward migration. Similar to other studies (Eisnecker, 2019), higher educational levels mean more positive attitudes toward migrants.

Therefore, the educational level becomes a vital aspect to consider as higher levels of education mean higher levels of conviviality, and thus, mitigates the levels of threat perceived with the presence of migrants. In other words, better education might allow making such (perceived) differences unremarkable—what Gilroy (2004) calls for when he refers to a convivial culture. However, in highly unequal segregated cities like Santiago, which has a greater concentration of migrants, income and educational levels go hand in hand, and thus we cannot know for certain if someone who has a higher educational level necessarily would be more convivial if they reside in multicultural neighborhoods, and where the presence of migrants is unavoidable. Furthermore, we need to acknowledge the fact that even achieving such ability to be convivial, from a nationally-based perspective implies that those who are “Othered” based on a *mestizo* normativity, in this case, are the ones who might carry the burden of conviviality (for instance, by suppressing their cultural norms, customs and habits) (see Redclift et al., 2022).

Regarding our contextual hypothesis (H3), while the proportion of migrants does not play a relevant role in the attitudes that were evaluated, changes in migration rates within territories can worsen the levels of conviviality. It is remarkable that greater increases in the migration rates mostly affect non-migrants' attitudes related to ensuring a *good coexistence* (conviviality) compared to the perception of threats related to identity and potential job loss. While the perception of international migrants as a threat in most national communities is not new, especially those in which national identity is a key part of people's identity formation, it is noteworthy that in times of crises, convivial attitudes tend to decrease in people who live in neighborhoods that have experienced major changes in migration rates.

Finally, we found partial evidence supporting our fourth hypothesis, which proposed that those non-migrants with lower status and who interact more with migrants would also tend to increase their negative attitudes toward them. Coincidently to these results, other studies have shown that working-class Chileans reproduce, foremost, anti-indigenous and, secondly anti-black racism through everyday practices and interactions in order to claim a white or whiter racial identity compared to LAC migrants (Bonhomme, 2022) and that the pandemic has reinforced an anti-immigrant sentiment, whereby Chileans perceive migrants' everyday practices as a threat to Chilean identity and customs (Bonhomme and Alfaro, 2022). Such negative perceptions do not take into account that what non-migrants conceive as migrants' cultural practices, which shape their forms of inhabiting, are only the inevitable outcome of the precarious housing conditions in which they are forced to live (Bonhomme, 2021). These negative perceptions of Peruvian and Venezuelan migrants seem to allow low-income Chileans to claim a higher social position in the social spectrum and assert racial superiority, especially due to the anti-indigenous racism that prevails against migrants from South American countries (Bonhomme, 2022). Similar to other ethnographic studies (2022), these results show that the need to mark a difference from other migrants with whom Chileans share similar ancestries (particularly the indigenous ancestry that is mostly acknowledged in the Chilean national identity, yet neglected by many as in the case of African ancestry), such as Peruvians and Venezuelans, becomes key for those Chileans with lower status, and who reside in low-income neighborhoods. Furthermore, as this study suggests, and in line with other research around the globe (Ahmad and Bradby, 2007; Cecchi, 2019), negative perceptions and stereotypes against migrants take greater force in times of disease outbreaks, putting social cohesion at risk. However, our results also reveal that the more contact and interaction non-migrants have with migrants is positively related with conviviality, so the fourth hypothesis is partially challenged. These results are aligned to other studies that show that intergroup friendship has a positive impact on attitudinal development (Davies et al., 2011; Hässler et al., 2019).

## Conclusions

This study attempted to be the first approach to the longitudinal changes in attitudes toward migration and their impact on different dimensions of social cohesion amid the COVID-19 pandemic in Chile. We were able to observe significant changes in different attitudinal dimensions, as an improvement in attitudes toward migrants in terms of conviviality and identity, and lower levels of threat during 2017 and 2018 (in reference to 2016) but with a prominent decay in conviviality and threat in the last survey wave (2021). These results are aligned with international and national studies that have shown that when societies face crises, people (and sometimes governments) tend to find scapegoats to blame, who are constructed as an “other” (Ahmad and Bradby, 2007; Cecchi, 2019; Bonhomme and Alfaro, 2022); in this case, LAC migrants. In that sense, Chileans would find it more difficult to live side by side with LAC migrants after times of crisis. The results give evidence about the increase of negative attitudes mostly by those with lower educational levels and with less contact with migrants, especially since 2020, which tend to be boosted by a larger proportion of LAC migrants in cities (see SJM, 2022).

It is interesting to note that the COVID-19 crisis mainly coincides with changes in attitudinal levels. We observed that the changes in Chileans' attitudes toward Peruvian and Venezuelan migrants over time had mainly to do with the fear of losing their jobs, and the threat to a constructed national identity and customs, and that it seems that the COVID-19 pandemic has worsened these negative perceptions. Nevertheless, although it would be tempting to attribute this phenomenon only to the COVID sanitary crisis and its implications on the economy, society, and culture over the globe, we are aware of several other processes that occur parallelly and that hinder the possibility to rule out different alternative explanations, such as the political turmoil in 2019 with the social outbreak (“*estallido social*”) and the consecutive changing scenarios. Some of these alternative explanations would be inflation, unemployment, and political instability, among others. Nonetheless, as some of these changes observed in 2020 were, at least in part, due to the sanitary crises, it makes it one of the key factors that allow us to better understand the radical changes we observed in this longitudinal study, many of which disrupted the improvements we saw over the years regarding the decrease of Chileans' negative attitudes toward the two migratory groups that were considered in the sample: Peruvians and Venezuelans. More research is needed in this regard, particularly the analysis of the changes in attitudes toward different migratory groups in subsequent waves of the survey panel data analyzed in this study.

It is vital to acknowledge that irregular migration grew significantly since 2018, which coincides with the significant changes in the migration policies that year. From a relatively increasing trend since 2012, the number of migrants crossing into Chile through irregular paths suddenly grew from 2,905 to 6,310 migrants by 2018 in a year and up to 8,048 in 2019. And when the pandemic hits, this number significantly increased to 16,848 in 2020 and then rose to 56,586 migrants in 2021, mainly coming from Venezuela (SJM, 2022). This opens up new debates on Chileans' attitudinal changes toward migrants and must be studied in detail in further research since it might influence more negative perceptions toward migrants (especially Venezuelans) from Chileans residing in increasingly multicultural neighborhoods.

Finally, while we acknowledge the theoretical discussions regarding the concept of conviviality and the need to approach it from all perspectives, we believe that in case studies where multiculturalism is still incipient yet steadily increasing in the Global South, a way to begin understanding these processes of multi-ethnic cohabitation using national surveys is from a nationally-based framework, since these samples are constituted by non-migrants. We understand however that the notion of conviviality needs to acknowledge the different processes that converge when approaching what would be a *friendly coexistence* or convivial multiculture (Back and Sinha, 2016), which, if achieved, might be usually at the expense of the constructed “other” within, in this case, a *mestizo* normativity and in a country that presumes a “raceless” character and has historically disavowed racism (Moreno Figueroa and Saldívar Tanaka, 2016; Bonhomme, 2022). In that sense, the research agenda, based on both quantitative and qualitative studies, should aim for understanding social cohesion and forms of multicultural coexistence over time, considering migrants' attitudes and perceptions of these processes of multi-ethnic cohabitation. Moreover, such research agenda should inform public policies about the consequences for social cohesion of high segregation and the lack of appropriate legal regulation of migration processes.

## Data availability statement

Publicly available datasets were analyzed in this study. This data can be found here: Panel survey study ELSOC, from the Centre for Social Conflict and Cohesion Studies https://coes.cl/encuesta-panel-manuales-metodologico-espanol/.

## Ethics statement

The studies involving human participants were reviewed and approved by the Scientific Ethics Committee of Social Sciences, Arts and Humanities of Pontificia Universidad Católica de Chile, June 8, 2016. The participants provided their written informed consent to participate in this study.

## Author contributions

All authors listed have made a substantial, direct, and intellectual contribution to the work and approved it for publication.

## Footnotes

^1^Latin American countries gave rise to new republics based on the former colonial “whiteness” hierarchies (Loveman, 2009). Like other countries of the Southern cone, Chile's nation state has taken whiteness for granted, upon the narrative of *mestizaje* that understand Chilean national identity as constituted solely by European-Spanish and indigenous ancestries (*mestizo)*, yet rejecting any African ancestry. This is due in part to the scant African presence as compared to other countries of the region, but foremost, to the historical rejection of the presence of Afro-Chileans, who still are not counted in the national Census. According to the first survey that characterized the Afro-descendant population in northern Chile, Arica (INE, 2015), 8,415 people self-identified as Afro-descendants, which would constitute a 0.05% of the total Chilean population if we consider the last Census. The state's *mestizaje* racial project meant a progressive whitening, assuming that such an intense racial mixture would dissolve the non-white ancestries (Goldberg, 2001; Bonhomme, 2022). Until these days, most Chileans, like their counterparts in other Southern cone countries (like Argentina), by self-identifying as *mestizos* they also feel “white” or at least “whiter” than other Latin Americans who are perceived as having more prevalent indigenous and/or African physical features.
